# The first complete mitochondrial DNA of the Chinese short-limbed skink (*Ateuchosaurus chinensis* Gray, 1845) determined by next-generation sequencing

**DOI:** 10.1080/23802359.2021.1891987

**Published:** 2021-03-18

**Authors:** Jun-Jie Zhong, Qian-Qian Wu, Yan-Mei Wang, Kun Guo, Guo-Hua Ding, Si-Te Luo

**Affiliations:** a Laboratory of Amphibian Diversity Investigation, College of Ecology, Lishui University, Lishui, Zhejiang, China; b College of Biology and the Environment, Nanjing Forestry University, Nanjing, Jiangsu, China; c College of Life and Environmental Science, Wenzhou University, Wenzhou, Zhejiang, China; d School of Life Sciences, Xiamen University, Xiamen, Fujian, China

**Keywords:** Scincidae, Ateuchosaurus chinensis Gray, 1845, mitochondrial DNA, phylogeny

## Abstract

The complete mitochondrial DNA (mtDNA) for the Chinese short-limbed skink (*Ateuchosaurus chinensis* Gray, 1845) was described by using next-generation sequencing. The total length of mtDNA was 16,840 bp, which contained 13 *PCGs* (*COI-III*, *ND1-6*, *ND4L*, *ATP6*, *ATP8*, and *CYTB*), 22 transfer RNA (*tRNA*) genes, 2 ribosomal RNA (*rRNA*) genes, and a control region (*D-loop*). The Bayesian inference tree showed that *A. chinensis* was a sister taxon to other scincid lizards in genera of *Scincella*, *Isopachys*, *Sphenomorphus* and *Tropidophorus*. The complete mtDNA of *A. chinensis* will be an important genetic resource to the studies of conservation and restoration of *A. chinensis*.

*Ateuchosaurus,* as an East Asian scincid genus, was composed of only two species, the Chinese short-limbed skink (*Ateuchosaurus chinensis* Gray, 1845) from southeastern China and northern Vietnam, and the Ryukyu short-legged skink (*A. pellopleurus* Hallowell, 1861) from the central and northern Ryukyus, Japan (Uetz et al. 2021). Previously, only the karyotypes of these two species have been reported (Ota et al. 1997). Recently, Makino et al. (2020) studied the before origin and intraspecific diversification of *A. pellopleurus* by molecular phylogeographic analyses, and found that the isolation of the two sister species was caused by a tectonic event in the Miocene. Here, we determined the complete mitochondrial DNA (mtDNA) of *A. chinensis* via next-generation sequencing, and compared the sequences with those of other scincid lizard species to analyze its phylogenetic placement.

One specimen of *A. chinensis* was captured from Xiafang Village (26.59196389°N, 116.92622491°E), Mingxi Country, Fujian Province, China, and then stored in 90% ethanol at the Museum of Laboratory of Amphibian Diversity Investigation (contact person: Guo-Hua Ding, E-mail: guwoding@lsu.edu.cn), Lishui University under the species voucher number LSU20200816GX01. Total genomic DNA was extracted from muscle tissue of *A. chinensis* using EasyPure Genomic DNA Kit (TransGen Biotech Co, Beijing, China). The whole genomic DNA library was fragmented with Covaris to an average insert size of 350 bp, and sequenced on the Illumina NovaSeq 6000 platform in Novogene Bioinformatics Technology Co. Ltd. (Tianjin, China). The complete mtDNA was assembled by *NOVO Plasty* 3.7 (Dierckxsens et al. 2017), based on the data of whole genomic sequencing.

The length of the *A. chinensis* complete mtDNA (GenBank accession: MW327509) was 16,840 bp, which contained 13 *PCGs* (*COI-III*, *ND1-6*, *ND4L*, *ATP6*, *ATP8*, and *CYTB*), 22 *tRNA* genes, 2 *rRNA* genes, and a control region (*D-loop*). We used *MITOS WebServer* (Bernt et al. 2013) and *tRNA-scan* (Chan and Lowe 2019) to determine the position and direction of these mtDNA genes. There are 9 genes (*tRNA^Gln^*, *tRNA^Ala^*, *tRNA^Asn^*, *tRNA^Cys^*, *tRNA^Tyr^*, *tRNA^Ser^*, *tRNA^Glu^*, *tRNA^Pro^*, and *NAD6*) on the minus chain, and the rest genes are on the plus chain. The longest *PCG* was 1,824 bp (*ND5*), but the shortest was 168 bp (*ATP8*). The length of *tRNAs* ranged from 64 bp to 75 bp. All *PCGs* initiated with ATG as a start codon, except for *COI* which began with GTG. 6 *PCGs* (*ND1*, *ND2*, *ND4L*, *ATP6*, *ATP8*, and *CYTB*) ended by TAA, 3 PCGs (*ND4*, *COII*, and *COIII*) were terminated with T, and the other 4 *PCGs* end with AGA (*COI*), TAG (*ND3*) and AGG (*ND5* and *ND6*) as the stop codons.

For determining the phylogenetic placement of *A. chinensis*, Bayesian inference (BI) tree was carried out in MrBayes v3.2.2 (Ronquist et al. 2012) using the parameters ‘ngen = 1,000,000 samplefreq = 1000 nchains = 4; mcmc; burnin = 1000’ based on 13 mitochondrial *PCGs* (11,406 bp) of 16 species from the superfamily Scincomorpha. After 1,000,000 generations, the run had converged when *p* < 0.01. *Lepidophyma flavimaculatum* Duméril, 1851 (Squamata: Xantusiidae) and *Smaug warreni* Boulenger, 1908 (Squamata: Cordylidae) were chosen as outgroups based on the report of Pyron et al. (2013). The best-fit substitution model with the smallest akaike information criterion was GTR + I + G calculated by MrModelTest 2.3 (Nylander 2004). Finally, the BI tree showed that *A. chinensis* formed a sister taxon to other scincid lizards in genera of *Scincella*, *Isopachys*, *Sphenomorphus* and *Tropidophorus* (Figure 1). The complete mtDNA of *A. chinensis* reported in this study will play an important role in understanding the evolution and systematic biology of the family Scincidae. Furthermore, it will be an important genetic resource to the studies of conservation and restoration of *A. chinensis.*

**Figure 1.**
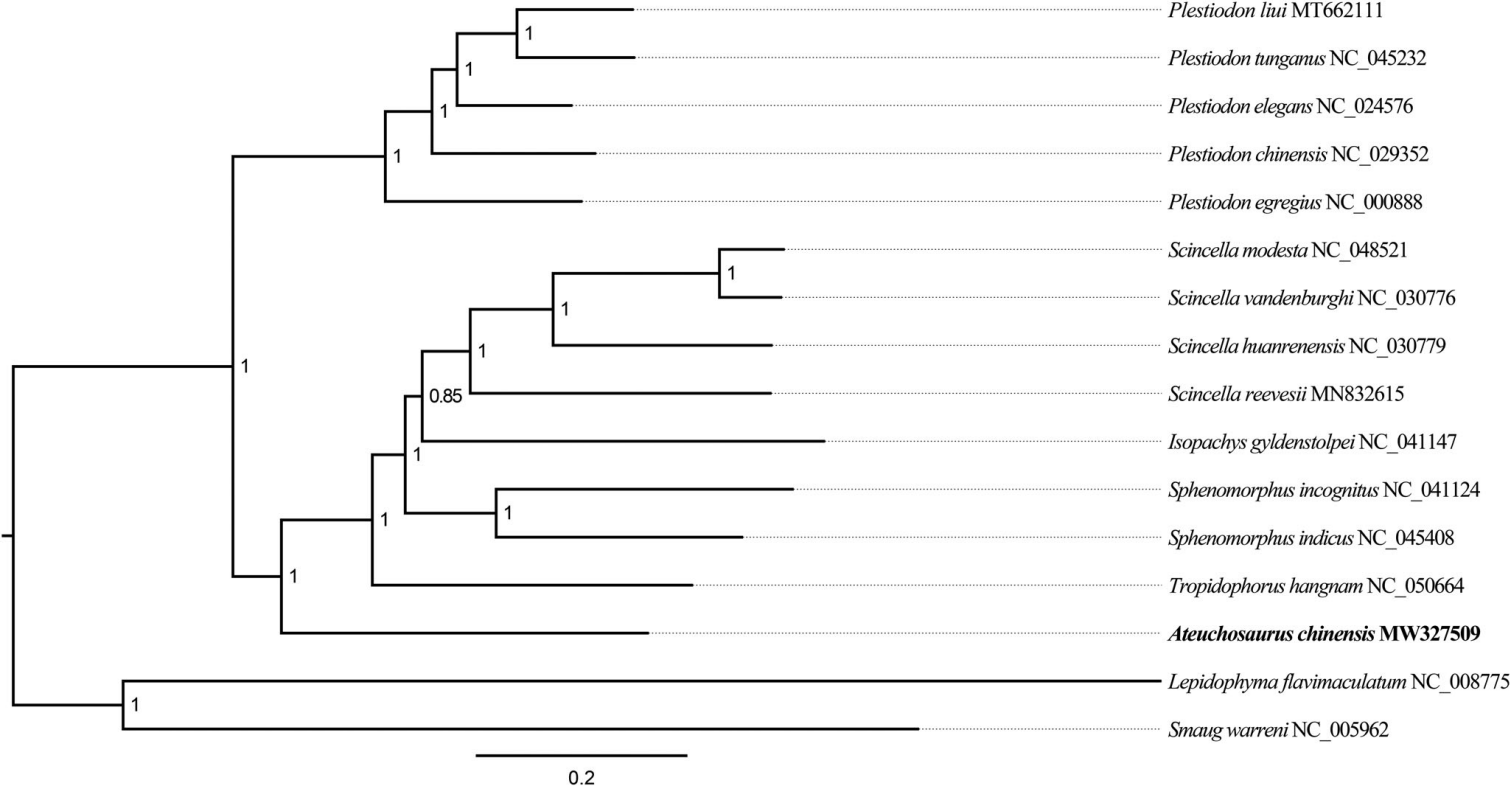
Bayesian inference phylogenetic tree inferred from mitochondrial DNA of superfamily Scincomorpha species based on an alignment of entire 13 *PCGs*. Support values (Bayesian posterior probabilities) were showed near the nodes.

## Data Availability

The mitogenome data supporting this study are openly available in GenBank at [https://www.ncbi.nlm.nih.gov/nuccore/MW327509]. Reference number [Accession number: MW327509]. BioSample and SRA accession numbers are [https://www.ncbi.nlm.nih.gov/biosample/SAMN17160231], [https://www.ncbi.nlm.nih.gov/sra/SRR13308285], respectively.
